# Feasibility and safety of single-port transvesical simple prostatectomy in patients with very large prostates: A volume-stratified analysis

**DOI:** 10.1038/s41391-026-01121-8

**Published:** 2026-05-28

**Authors:** Jin-Chun Qi, Sheng Tang, Dong-Bin Wang, Ze-Lin Fu, Lorenzo Santodirocco, Marwan Alkassis, Ruben Sauer Calvo, Chang-Bao Qu, Ya-Xuan Wang, Simone Crivellaro

**Affiliations:** 1 Department of Urology, The Second Hospital of Hebei Medical University, No.215 Heping West Road, Shijiazhuang, China; 2 Department of Urology, University of Illinois at Chicago, Chicago, IL USA; 3 Department of Maternal-Infant and Urological Sciences, Sapienza University of Rome, Umberto I Hospital, Rome, Italy; 4 Department of Urology, Shijiazhuang People’s Hospital, Shijiazhuang, 050011 China

**Keywords:** Prostatic diseases, Outcomes research

## Abstract

**Purpose:**

Single-port transvesical robot-assisted simple prostatectomy (SP-SP) is a minimally invasive treatment option for patients with large prostates. However, the impact of prostate volume on perioperative outcomes, particularly in patients with extremely large glands, has not been systematically evaluated. This study aimed to assess the safety and efficacy of SP-SP across different prostate volume strata.

**Materials and methods:**

We retrospectively reviewed 90 consecutive patients who underwent SP-SP between 2020 and 2025 by a single surgeon. Patients were stratified by preoperative total prostate volume (TPV) into Group A (80–120 mL, *n* = 43), Group B (120–160 mL, *n* = 22), and Group C (>160 mL, *n* = 25). Perioperative parameters were compared among the three groups. Multivariate logistic regression analysis was performed to identify risk factors for postoperative complications.

**Results:**

Baseline characteristics were generally comparable among groups. Pairwise comparisons showed that patients in Group C had significantly higher preoperative PSA levels than those in Group A, as well as longer operative time and greater estimated blood loss (EBL) compared with both Groups A and B. Aside from postoperative hemoglobin levels, recovery and safety outcomes were largely similar across groups. Multivariate analysis identified EBL as the only independent risk factor for postoperative complications, while TPV was not independently associated with complications. Specimen weight increased with increasing prostate volume, while the resection ratio was similar across groups. At the last follow-up, significant improvements were observed in PSA, PVR, IPSS, and QoL scores, as well as in the urinary continence across the three groups, with no significant differences between groups.

**Conclusions:**

These findings provide preliminary evidence supporting the feasibility of SP-SP in patients with very large prostates. Larger multicenter studies with adequate sample sizes and longer follow-up are warranted to further evaluate these results.

## Introduction

Benign prostatic hyperplasia (BPH) is a major cause of lower urinary tract symptoms (LUTS) in middle-aged and older men and can significantly impair voiding function and quality of life. Surgery remains the mainstay of treatment for patients who fail medical therapy or develop complications such as recurrent urinary retention, hematuria, bladder stones, or upper urinary tract dilation. In patients with large prostates, prior studies have suggested that simple prostatectomy or laser enucleation of the prostate (LEP) is the preferred surgical option when prostate volume exceeds 80 mL [1]. Among these approaches, robot-assisted simple prostatectomy (RASP) has emerged as an important minimally invasive alternative to open surgery because it allows effective adenoma removal while reducing perioperative blood loss, shortening hospitalization, and facilitating postoperative recovery [2–4].

With the development of the da Vinci single-port (SP) platform, single-port transvesical robot-assisted simple prostatectomy (SP-SP) has further advanced the minimally invasive management of large-volume BPH. Through a single suprapubic incision, this technique accesses the bladder directly, allowing enucleation of the adenoma within a confined surgical field. Theoretical advantages include avoidance of transperitoneal dissection, less postoperative pain, shorter hospitalization, and allowing concomitant bladder pathology during the same procedure [2, 5]. In a clinical series of 117 patients, Ramos et al. reported that SP-SP was completed successfully in patients with large prostates without the need for additional ports or conversion to open surgery; most patients were discharged within 24 h and experienced sustained improvement in urinary function [5]. Subsequent multicenter experience also demonstrated low complication rates, short catheterization times, and rapid recovery across different surgeons and institutions [6]. Recent functional outcome studies with longer follow-up have further suggested favorable continence recovery, a low incidence of postoperative incontinence, sustained improvement in LUTS, and stable long-term continence and sexual function after the procedure [3, 7].

However, the current evidence on SP-SP remains largely focused on procedural description, early experience, overall perioperative outcomes, functional recovery, and technical feasibility [4–7]. By contrast, prostate volume has been recognized as an important factor influencing surgical complexity and perioperative outcomes in prostate surgery, yet its specific impact on perioperative safety, intraoperative burden, and postoperative recovery after SP-SP has not been systematically evaluated in a stratified analysis [8]. This question is particularly relevant when gland size exceeds 120 mL, and even more so when it exceeds 160 mL, because exposure, identification of the enucleation plane, hemostasis, and reconstruction may all become more demanding. Our group recently showed that prostate volume is an independent determinant of operative performance in single-port transvesical RASP and has a greater impact on operative time than body mass index (BMI) [8]. Therefore, we performed a single-center study of consecutive patients stratified by preoperative total prostate volume (TPV) to assess the impact of prostate size on perioperative outcomes after SP-SP and to better define the clinical performance of this technique in patients with very large prostates.

## Subjects and methods

### Study design and population

This was a single-center, retrospective cohort study including consecutive patients who underwent SP-SP between January 2020 and December 2025 at the University of Illinois Medical Center in Chicago, Illinois, USA, a tertiary referral center. All procedures were performed by a single attending surgeon with more than 10 years of experience in minimally invasive surgery and more than 100 prior cases of multi-port RASP. The study cohort partially overlapped with our previously published learning curve study, and additional consecutive cases performed thereafter were also included. Learning-curve phases were defined according to our analysis and categorized as learning, proficiency, and consolidation phases. Due to differences in inclusion criteria, the final sample size differed from that of the previously reported cohort. Inclusion criteria were a prostate volume of at least 80 mL on preoperative transabdominal ultrasonography or magnetic resonance imaging, moderate to severe LUTS with recurrent urinary retention, hematuria, bladder stones, and hydronephrosis. Exclusion criteria included prior prostate surgery, concomitant prostate cancer requiring radical prostatectomy, a history of major bladder surgery, and neurogenic bladder dysfunction.

### Group stratification

Patients were stratified according to preoperative imaging-based total prostate volume (TPV) into three groups: Group A (80 mL ≤ TPV < 120 mL), Group B (120 mL ≤ TPV < 160 mL), and Group C (TPV ≥ 160 mL). Currently, there is no universally accepted definition of “large” prostate volume. The European Association of Urology generally defines prostates >80 ml as large [9]. In this study, the 120 mL and 160 mL cutoffs were prespecified prior to analysis. The 120 mL threshold was selected because, in our institutional practice, glands exceeding approximately 120 g typically require division of the adenoma into two or three parts during dissection to facilitate controlled enucleation within the confined single-port operative field, as described in Section 2.3. The 160 mL threshold is broadly consistent with commonly used definitions of very large prostates (>150 mL) in the literature [10] and allowed for a reasonable distribution of sample sizes across groups, thereby improving the stability of between-group comparisons.

### Surgical procedure

The methodology for transvesical SP-RASP has been previously described [8, 11]. Briefly, after establishing transvesical access, the ureteral orifices were identified and the enucleation plane was delineated. The adenoma was enucleated anteriorly at the bladder neck and progressed toward the prostatic apices with careful preservation of the ureteral orifices.

In cases of massive prostatic enlargement, significant anatomical displacement of the bladder neck and trigone limits the triangulation space within the single-port setup. For glands exceeding approximately 120 g, the adenoma was divided into two or three parts during dissection, depending on whether the anatomy was bilobar or trilobar, to facilitate controlled enucleation within the confined operative field. When a prominent median lobe obstructed visualization of the posterior plane, a median lobectomy was performed first. For bulky lateral lobes restricting instrument excursion, the adenoma was partitioned along anatomical grooves and enucleated in a subtotal lobar fashion, followed by additional clearance of the capsular surface to ensure complete resection. Large adenomas typically displace the bladder neck cranially and ventrally, resulting in excessive anastomotic tension. To address this, posterior dissection was extended deep to Denonvilliers’ fascia, occasionally exposing the tail of the seminal vesicles, to achieve adequate mobilization and tension-free descent of the bladder neck.

Following urethral transection, intravesical pressure was reduced to facilitate hemostasis. The reconstruction process involved advancing the posterior mucosa without completing a circumferential vesicourethral anastomosis, thereby preserving anatomical integrity and optimizing recovery. The procedure concluded with catheter placement and bladder closure.

### Statistical analysis

Statistical analyses were performed using R software (version 4.5.1). The variables included in the analysis were: baseline characteristics—age, race, BMI, TPV, age-adjusted Charlson Comorbidity Index (age-adjusted CCI), American Society of Anesthesiologists (ASA) classification and the learning curve phase; perioperative parameters—prostate-specific antigen (PSA) level, hemoglobin values, postvoid residual volume (PVR), International Prostate Symptom Score (IPSS), quality of life (QoL) score, indwelling urinary catheter status, history of prior abdominal surgery, urine culture results, estimated blood loss (EBL), operative time, intraoperative opioid dosage and performance of bladder irrigation, postoperative pain and analgesic requirements, duration of catheterization, length of hospital stay and Clavien–Dindo complications; pathological data—specimen weight, resection ratio (specimen weight / preoperative TPV), postoperative pathological type, and Gleason score; and follow-up measures—PSA level, PVR, IPSS, and QoL scores at the last follow-up, and postoperative urinary continence status. EBL was calculated as the total suction canister volume after subtraction of the irrigation fluid instilled during the procedure. To reduce potential contamination from urinary output, the bladder was routinely drained immediately after transvesical access and before adenoma enucleation, and continuous bladder irrigation was not routinely employed. Given the transvesical approach, some degree of imprecision due to ongoing urine production is unavoidable. The primary endpoints were operative time, EBL, and postoperative complications. Secondary endpoints included perioperative recovery parameters, functional outcomes, and pathological outcomes.

Continuous variables were presented as medians with interquartile ranges (IQRs). Comparisons among the three groups were performed using the Kruskal-Wallis test. When the overall comparison yielded statistical significance, post hoc pairwise comparisons were conducted using the Dunn test. Categorical variables were summarized as frequencies and percentages. Comparisons between groups were performed using the chi-square test or Fisher’s exact test, as appropriate. To account for potential confounding related to the surgical learning curve, a sensitivity analysis was performed by excluding patients in the learning phase, and study outcomes were re-evaluated in the remaining cohort. Additionally, multivariate logistic regression analysis was performed to identify risk factors for postoperative complications in this cohort. In this model, TPV was analyzed as a continuous variable (per 10 mL increase) to avoid potential biases introduced by arbitrary volume thresholds and to assess whether prostate volume exerts an independent, dose-dependent effect on the risk of postoperative complications. Results were reported as odds ratios (ORs) with 95% confidence intervals (CIs). A two-tailed *P*-value < 0.05 was considered to indicate statistical significance.

## Results

### Baseline characteristics

A total of 90 patients were included and stratified by preoperative TPV into Group A (*n* = 43), Group B (*n* = 22), and Group C (*n* = 25). Median TPV values were 93.0 (83.0–100.0) mL in Group A, 130.0 (120.0–138.0) mL in Group B, and 240.0 (180.0–290.0) mL in Group C. No significant between-group differences were observed in age, race, BMI, age-adjusted CCI, ASA classification or learning-curve phase distribution (all *P* > 0.05) (Table 1). In a sensitivity analysis excluding the initial learning phase, median prostate volumes increased slightly across all groups, while baseline characteristics remained well balanced (Supplementary Table S1).

**Table 1:** Baseline characteristics of patients in different prostate volume groups.

Characteristics	Prostate volume group			*p* value
	Group A (80–120 mL) (*N* = 43)	Group B (120–160 mL) (*N* = 22)	Group C (>160 mL) (*N* = 25)	
Age, Median (IQR) (years)	43 | 64.0 (59.0, 71.0)	22 | 65.0 (61.0, 70.0)	25 | 67.0 (65.0, 73.0)	0.169
Race, n (%)				0.073
African American	15 (34.9%)	9 (40.9%)	12 (48.0%)	
Asian	2 (4.7%)	0 (0.0%)	0 (0.0%)	
Caucasian	5 (11.6%)	2 (9.1%)	8 (32.0%)	
Hispanic	21 (48.8%)	11 (50.0%)	5 (20.0%)	
BMI, Median (IQR) (kg/m^2^)	43 | 27.7 (24.4, 30.1)	22 | 28.2 (25.8, 30.7)	25 | 29.8 (23.8, 37.3)	0.364
Prostate size, Median (IQR) (mL)	43 | 93.0 (83.0, 100.0)	22 | 130.0 (120.0, 138.0)	25 | 240.0 (180.0, 290.0)	**<0.001**
Age adjusted CCI, n (%)				0.992
0–3	31 (72.1%)	16 (72.7%)	17 (68.0%)	
4–5	10 (23.3%)	5 (22.7%)	6 (24.0%)	
≥6	2 (4.7%)	1 (4.5%)	2 (8.0%)	
ASA grade, n (%)				0.090
2	19 (44.2%)	12 (54.5%)	6 (24.0%)	
3	24 (55.8%)	10 (45.5%)	19 (76.0%)	
Learning curve phase, n (%)				0.731
Learning phase	12 (27.9%)	9 (40.9%)	6 (24.0%)	
Proficiency phase	17 (39.5%)	7 (31.8%)	12 (48.0%)	
Consolidation phase	14 (32.6%)	6 (27.3%)	7 (28.0%)	

*BMI* body mass index, *CCI* Charlson comorbidity index, *ASA* American Society of Anesthesiologists. Bold values indicate statistical significance with P < 0.05.

### Preoperative characteristics

Preoperative clinical characteristics are shown in Table 2. Overall, the preoperative profiles were comparable across groups. Preoperative PSA increased across increasing TPV strata and differed significantly among groups (overall *P* = 0.003). Pairwise comparisons showed that PSA levels were significantly elevated in Group C compared with Group A (*P* = 0.002). We observed a higher rate of positive preoperative urine cultures in Group C (44%) than in Group A (27.9%) and Group B (27.3%), although this difference did not reach statistical significance. Sensitivity analysis yielded consistent findings: preoperative PSA remained higher in Group C than in Group A (*P* = 0.021), while all other preoperative parameters remained comparable across the three groups (Supplementary Table S2).

**Table 2:** Preoperative characteristics of patients in different prostate volume groups.

Characteristics	Prostate volume group			*p* value
	Group A (80–120 mL) (*N* = 43)	Group B (120–160 mL) (*N* = 22)	Group C (>160 mL) (*N* = 25)	
Preoperative PSA, (ng/mL)	31 | 4.6 (2.3, 9.7)	19 | 8.8 (3.3, 12.8)	20 | 12.0 (5.2, 22.9)	**0.003**
Median (IQR)				
Preoperative hemoglobin, (g/dL)	39 | 13.7 (12.9, 14.8)	21 | 13.5 (12.7, 14.7)	22 | 14.1 (12.1, 14.5)	0.764
Median (IQR)				
Preoperative PVR, (ml)	36 | 110.0 (52.0, 304.0)	15 | 230.0 (45.0, 600.0)	19 | 155.0 (73.0, 600.0)	0.504
Median (IQR)				
Preoperative IPSS	27 | 23.0 (20.0, 27.0)	15 | 20.0 (14.0, 25.0)	11 | 18.0 (15.0, 27.0)	0.179
Median (IQR)				
Preoperative QoL	28 | 5.0 (4.0, 5.0)	18 | 4.0 (3.0, 5.0)	13 | 4.0 (3.0, 4.0)	0.171
Median (IQR)				
Preoperative indwelling catheter, n (%)				0.203
No	27 (62.8%)	9 (40.9%)	12 (48.0%)	
Yes	16 (37.2%)	13 (59.1%)	13 (52.0%)	
Preoperative prostate medication, n (%)				0.795
5α-Reductase inhibitor	1 (2.3%)	1 (4.5%)	0 (0.0%)	
Alpha Blocker	24 (55.8%)	9 (40.9%)	13 (52.0%)	
Alpha Blocker + 5α-Reductase inhibitor	13 (30.2%)	9 (40.9%)	9 (36.0%)	
Anticholinergic	0 (0.0%)	1 (4.5%)	0 (0.0%)	
None	5 (11.6%)	2 (9.1%)	3 (12.0%)	
Prior abdominal surgery, n (%)				0.720
No	35 (81.4%)	16 (72.7%)	19 (76.0%)	
Yes	8 (18.6%)	6 (27.3%)	6 (24.0%)	
Preoperative prostate biopsy, n (%)				0.141
No	30 (71.4%)	11 (50.0%)	13 (52.0%)	
Yes	12 (28.6%)	11 (50.0%)	12 (48.0%)	
Preoperative urine culture, n (%)				0.362
Negative	31 (72.1%)	16 (72.7%)	14 (56.0%)	
Positive	12 (27.9%)	6 (27.3%)	11 (44.0%)	

*PSA* prostate-specific antigen, *PVR* postvoid residual volume, *IPSS* International Prostate Symptom Score, *QoL* quality of life, *MME* morphine milligram equivalents. Bold values indicate statistical significance with P < 0.05.

### Intraoperative outcomes

Operative time and EBL both differed significantly among the three groups (overall *P* < 0.001) (Table 3). Pairwise comparisons revealed that operative time was significantly longer in Group C than in Group A (*P* < 0.001) and Group B (*P* = 0.024), whereas no significant difference was observed between Groups A and B (*P* = 0.372). Similarly, EBL was higher in Group C than in Group A (*P* < 0.001) and Group B (*P* = 0.036), with no significant difference between Groups A and B (*P* = 0.815). Sensitivity analysis confirmed the overall pattern, with operative time and EBL remaining greater in Group C than in Group A and Group B (both *P* < 0.05). Notably, median operative times decreased across all groups following this exclusion (Supplementary Table S3).

**Table 3:** Intraoperative outcomes of patients in different prostate volume groups.

Characteristics	Prostate volume group			*p* value
	Group A (80–120 mL) (*N* = 43)	Group B (120–160 mL) (*N* = 22)	Group C (>160 mL) (*N* = 25)	
Operative time, (minutes)	43 | 181.0 (147.0, 202.0)	22 | 190.0 (172.0, 220.0)	25 | 227.0 (196.0, 275.0)	**<0.001**
Median (IQR)				
Estimated blood loss, (mL)	43 | 50.0 (30.0, 130.0)	22 | 65.0 (50.0, 200.0)	25 | 200.0 (130.0, 200.0)	**<0.001**
Median (IQR)				
Intraoperative opioid dose, (mg MME)	42 | 12.5 (12.0, 15.0)	20 | 14.0 (7.4, 17.0)	24 | 14.3 (12.3, 17.8)	0.427
Median (IQR)				
Bladder irrigation, n (%)				0.397
No	41 (95.3%)	19 (86.4%)	22 (91.7%)	
Yes	2 (4.7%)	3 (13.6%)	2 (8.3%)	

Bold values indicate statistical significance with P < 0.05.

### Postoperative outcomes

Recovery and safety profiles were broadly comparable across groups (Table 4). Postoperative hemoglobin levels differed among the three groups (overall *P* = 0.014), with pairwise comparisons revealing lower values in Group B than in Group A (P = 0.046). The hemoglobin drop showed a stepwise increase from Group A to Group C, though this trend did not reach statistical significance (*P* = 0.087). In the sensitivity analysis, the intergroup difference in postoperative hemoglobin was no longer statistically significant (*P* = 0.071), while the stepwise increase in hemoglobin drop persisted without reaching significance (*P* = 0.276) (Supplementary Table S4). Notably, compared with Groups A and B, Group C exhibited a longer hospital stay, a longer catheterization time, and a lower same-day discharge rate. However, these differences were not statistically significant. No significant differences were observed among the three groups regarding overall complication rates or the distribution of Clavien-Dindo grades (all *P* > 0.05). All complications were below grade III. Most adverse events were genitourinary in nature. One patient in Group C developed sepsis and responded well to conservative antibiotic treatment.

**Table 4:** Postoperative outcomes of patients in different prostate volume groups.

Characteristics	Prostate volume group			*p* value
	Group A (80–120 mL) (*N* = 43)	Group B (120–160 mL) (*N* = 22)	Group C (>160 mL) (*N* = 25)	
Postoperative hemoglobin (g/dL)	24 | 13.1 (11.7, 14.5)	13 | 11.1 (10.0, 13.0)	12 | 11.5 (9.7, 12.6)	**0.014**
Median (IQR)				
Hemoglobin drop (g/dL)	24 | 0.5 (−0.1, 1.7)	13 | 1.0 (0.4, 2.7)	12 | 1.7 (0.6, 3.0)	0.087
Median (IQR)				
Pain score at postoperative	43 | 3.0 (0.0, 4.0)	22 | 3.0 (0.0, 4.0)	25 | 3.0 (0.0, 4.0)	0.920
Median (IQR)				
Postoperative opioid dose, (mg MME)	41 | 7.5 (5.0, 10.0)	20 | 2.5 (0.0, 13.3)	22 | 6.0 (0.0, 10.0)	0.350
Median (IQR)				
Total opioid dose on POD 0, (mg MME)	41 | 20.0 (12.5, 27.0)	20 | 19.8 (12.9, 25.8)	22 | 21.0 (17.5, 24.0)	0.749
Median (IQR)				
Time to catheter removal, (days)	43 | 5.0 (4.0, 6.0)	21 | 5.0 (4.0, 6.0)	25 | 6.0 (4.0, 7.0)	0.133
Median (IQR)				
Length of hospital stay, (IQR) (hours)	43 | 9.0 (8.0, 10.0)	21 | 9.0 (9.0, 10.0)	25 | 11.0 (9.0, 25.0)	0.058
Same Day Discharge, n (%)				0.067
No	7 (16.3%)	3 (14.3%)	10 (40.0%)	
Yes	36 (83.7%)	18 (85.7%)	15 (60.0%)	
OFA, n (%)				0.118
No	32 (78.0%)	11 (55.0%)	13 (59.1%)	
Yes	9 (22.0%)	9 (45.0%)	9 (40.9%)	
Analgesia at discharge, n (%)				0.724
Opioid	1 (2.3%)	0 (0.0%)	0 (0.0%)	
No opioid	29 (67.4%)	16 (76.2%)	17 (70.8%)	
Association	8 (18.6%)	2 (9.5%)	6 (25.0%)	
No	5 (11.6%)	3 (14.3%)	1 (4.2%)	
Postoperative complications, n (%)				0.442
No	31 (72.1%)	14 (63.6%)	20 (80.0%)	
Yes	12 (27.9%)	8 (36.4%)	5 (20.0%)	
Clavien-Dindo grade, n (%)				0.864
1	5 (41.7%)	2 (25.0%)	2 (40.0%)	
2	7 (58.3%)	6 (75.0%)	3 (60.0%)	
Readmission, n (%)				0.082
No	43 (100.0%)	20 (90.9%)	24 (96.0%)	
Yes	0 (0.0%)	2 (9.1%)	1 (4.0%)	
Postoperative urine culture, n (%)				0.607
Negative	25 (71.4%)	15 (83.3%)	14 (82.4%)	
Positive	10 (28.6%)	3 (16.7%)	3 (17.6%)	

*POD* postoperative day, *MME* morphine milligram equivalents, *OFA* opioid-free analgesia. Bold values indicate statistical significance with P < 0.05.

### Pathological and follow-up outcomes

Pathological and follow-up outcomes are summarized in Table 5. Specimen weight increased with TPV, with all pairwise comparisons showing statistically significant differences among the three groups. Interestingly, the median resection ratio was slightly lower in Group C (57.0%) than in Group A (61.0%) and Group B (63.5%), although this difference did not reach statistical significance (*P* = 0.414). In the sensitivity analysis, median resection ratios converged across the three groups (61.0%, 62.0%, and 60.0%, respectively; *P* = 0.683) (Supplementary Table S5). The incidence of incidental prostate cancer on pathology did not differ across groups (P = 0.723). Gleason score distribution was as follows: Group A, 2 cases of Gleason 6 and 2 cases of Gleason 7; Group B, 1 case of Gleason 7; and Group C, 1 case of Gleason 6 and 2 cases of Gleason 7. At the last follow-up, no significant differences were observed among the groups in PSA, PVR, IPSS, or QoL scores (all *P* > 0.05). The continence recovery rates at 3 months postoperatively were 97.7%, 95.5%, and 91.7% in Groups A, B, and C, respectively (*P* = 0.572). By 6 months, all patients had achieved complete continence in all three groups.

**Table 5:** Pathological and follow-up outcomes of patients in different prostate volume groups.

Characteristics	Prostate volume group			*p* value
	Group A (80–120 mL) (*N* = 43)	Group B (120–160 mL) (*N* = 22)	Group C (>160 mL) (*N* = 25)	
Specimen weight, (g)	43 | 55.0 (42.0, 69.0)	22 | 83.0 (74.0, 92.0)	25 | 138.0 (116.0, 176.0)	**<0.001**
Median (IQR)				
Resection ratio, (%)	43 | 61.0 (45.0, 69.0)	22 | 63.5 (57.0, 71.0)	25 | 57.0 (53.0, 71.0)	0.414
Median (IQR)				
Postoperative pathology, n (%)				0.723
BPH	39 (90.7%)	21 (95.5%)	22 (88.0%)	
Cancer	4 (9.3%)	1 (4.5%)	3 (12.0%)	
PSA at last follow-up, (ng/mL)	19 | 0.6 (0.2, 1.0)	12 | 0.3 (0.1, 0.6)	15 | 0.6 (0.3, 1.0)	0.215
Median (IQR)				
PVR at last follow-up, (ml)	40 | 8.5 (0.0, 22.5)	20 | 8.5 (0.0, 48.0)	23 | 9.0 (0.0, 20.0)	0.795
Median (IQR)				
Postoperative IPSS	34 | 4.0 (3.0, 5.0)	16 | 5.0 (2.0, 6.5)	18 | 5.0 (3.0, 7.0)	0.329
Median (IQR)				
Postoperative QoL	33 | 1.0 (1.0, 2.0)	18 | 1.0 (0.0, 2.0)	13 | 1.0 (0.0, 1.0)	0.766
Median (IQR)				
Continence in 3 months, n (%)				0.572
No	1 (2.3%)	1 (4.5%)	2 (8.3%)	
Yes	42 (97.7%)	21 (95.5%)	22 (91.7%)	
Continence in 6 months, n (%)				1.000
No	0 (0.0%)	0 (0.0%)	0 (0.0%)	
Yes	32(100%)	15(100%)	16(100%)	

*PSA* prostate-specific antigen, *PVR* postvoid residual volume, *IPSS* International Prostate Symptom Score, *QoL* quality of life, *BPH* benign prostatic hyperplasia. Bold values indicate statistical significance with P <0.05.

### Multivariate logistic analysis for postoperative complications

The results of the multivariate logistic regression analysis are presented in Table 6. EBL was identified as an independent risk factor for postoperative complications (OR = 1.07, 95% CI: 1.01–1.15, *P* = 0.030), with each 10 mL increment conferring a 7% increase in complication risk. No significant associations were observed for prostate volume (OR = 0.96, *P* = 0.347), operative time (OR = 0.96, *P* = 0.606), or any phase of the learning curve.

**Table 6:** Multivariate logistic regression analysis of risk factors for postoperative complications.

Variable	OR (95% CI)	*P* value
Prostate volume (per 10 mL increase)	0.96 (0.88–1.04)	0.347
Estimated blood loss (per 10 mL increase)	1.07 (1.01–1.15)	**0.030**
Operative time (per 10 min increase)	0.96 (0.84–1.10)	0.606
Learning curve (ref: Learning phase)		
Proficiency phase	1.29 (0.35–4.93)	0.702
Consolidation phase	1.18 (0.32–4.38)	0.806

Bold values indicate statistical significance with P <0.05.

## Discussion

To the best of our knowledge, this is the first study to evaluate the impact of prostate volume stratification on perioperative outcomes following SP-SP, with a specific focus on patients with very large prostates. Our findings suggest that increasing prostate volume affects SP-SP mainly by increasing intraoperative complexity, without a clear adverse effect on perioperative safety or short- to intermediate-term functional outcomes. Apart from a progressive increase in PSA with increasing gland size, no clinically meaningful differences were observed in preoperative characteristics. In the present cohort, larger prostates were associated with longer operative time and higher EBL, while a gradual volume-outcome relationship remains likely. Importantly, postoperative pain scores, time to catheter removal, length of stay, readmission, and overall complication rates did not differ significantly, and all complications were below Clavien-Dindo grade III, supporting the overall safety of SP-SP even in very large prostates. Multivariate logistic regression further demonstrated that prostate volume was not independently associated with postoperative complications, whereas EBL was the only independent risk factor. Notably, the lowest absolute postoperative hemoglobin level was observed in Group B rather than Group C. This finding does not necessarily indicate greater true blood loss in Group B. Postoperative hemoglobin is influenced not only by intraoperative bleeding but also by baseline hemoglobin levels, perioperative fluid resuscitation, hemodilution, timing of blood sampling, and the limited number of available measurements. Although also subject to these confounding factors, hemoglobin decline increased stepwise from Groups A to C and showed a similar trend to intraoperative EBL; therefore, it should be interpreted with caution. Specimen weight increased significantly across volume strata. Interestingly, the median resection ratio was numerically lower in Group C (57.0%) than in Groups A (61.0%) and B (63.5%), although this difference was not statistically significant. This trend was no longer evident in the sensitivity analysis excluding the learning phase (61.0%, 62.0%, and 60.0%, respectively), likely reflecting more conservative enucleation during the early learning curve. In addition, the higher rate of preoperative positive urine cultures in Group C (44.0%) may have introduced measurement bias. Active infection can lead to tissue edema, potentially resulting in overestimation of imaging-based prostate volume. Because specimen weight was measured as dry weight ex vivo, this discrepancy may partially explain the lower resection ratio observed in this group. At the last follow-up, all groups showed substantial improvements in PSA, PVR, IPSS, and QoL scores, as well as in continence recovery at 3 and 6 months, with no significant between-group differences. All patients had achieved complete continence by 6 months. Overall, these findings indicate that although SP-SP in patients with larger prostates is associated with longer operative duration and greater blood loss, it does not appear to increase major complications or compromise postoperative recovery and functional benefit.

These findings are broadly consistent with the literature on surgery for large-volume prostates, while further clarifying outcomes with a single-port transvesical approach. Open simple prostatectomy (OSP) was historically the standard treatment for prostates >80 mL, but at the cost of greater trauma, blood loss, and hospitalization. Systematic reviews and meta-analyses have shown that, although RASP may require longer operative time than OSP, it is associated with lower blood loss, lower transfusion rates, shorter hospitalization, and fewer overall complications, with broadly similar functional outcomes [12–14]. Prior studies have also shown that robot-assisted simple prostatectomy provides effective debulking and acceptable perioperative safety in large glands, but evidence stratified by prostate volume remains limited, especially for extremely enlarged prostates. Morozov et al. reported that RASP is generally safe and effective with respect to both subjective and objective voiding outcomes, while also highlighting the need for subgroup analyses across volume ranges [10]. With respect to SP-SP, prior single-center, multi-institutional, and functional-outcome studies have shown low blood loss, rapid recovery, short hospitalization, and favorable postoperative voiding and continence outcomes [2–7, 15–17]. Current meta-analytic evidence further indicates that, relative to endoscopic enucleation procedures, RASP may not be superior in terms of hemoglobin decline, transfusion rate, catheter duration, or length of stay, although functional outcomes and most complications are generally comparable [12, 18]. Accordingly, the value of SP-SP may lie not in universal superiority, but in offering a minimally invasive option for extremely large prostates that preserves effective debulking with acceptable perioperative safety.

Clinically, these findings suggest that larger prostate volume is associated with increased intraoperative complexity during SP-SP. As gland size increases, the adenoma more often protrudes into the bladder and outlet, the dissection plane between adenoma and capsule becomes longer and less regular, and vascularity is often richer [19, 20]. These factors likely make enucleation, hemostasis, and bladder mucosal reconstruction more difficult, which may explain the significantly longer operative time and greater EBL in group C. At the same time, the larger gland burden in our cohort was not accompanied by higher rates of severe complications or clearly worse postoperative functional outcomes. This pattern may be related, at least in part, to the technical features of the approach. The transvesical single-port route allows surgery within a confined bladder cavity and avoids extensive intraperitoneal dissection. Pneumovesicum pressure, direct visualization of the prostate, and advancement or reconstruction of bladder mucosa may facilitate hemostasis, coverage of the raw surface, and stabilization of the bladder neck, potentially reducing uncontrolled bleeding and bladder neck-related complications [3, 13, 14, 17]. In addition, the SP platform retains articulation and suturing capability despite the single-port setting, and prospective platform studies have suggested that it is clinically feasible without a major ergonomic disadvantage [21, 22]. These observations support the view that, in experienced hands, extremely large prostates primarily increase technical workload rather than perioperative risk. Overall, our data support SP-SP as a reasonable minimally invasive option for selected patients with extremely large prostates [12, 13, 17].

This study has several limitations. It was retrospective, single-center, and based on a single-surgeon experience, which reduces inter-surgeon variability but limits generalizability. The sample size was modest, particularly in the >160 mL group, which may have reduced statistical power. Although operative time was significantly longer in the largest-volume group, the retrospective design limited the availability of detailed step-specific operative metrics (e.g., access establishment, adenoma enucleation, hemostasis, and reconstruction), precluding a more granular analysis of procedural complexity. Missing data were present for several postoperative functional variables and follow-up assessments (e.g., postoperative hemoglobin and hemoglobin drop were missing in >50% of patients; PSA at last follow-up, postoperative IPSS, and postoperative QoL were missing in approximately 25–50% of patients), and follow-up time points were not fully standardized. Although the proportion of missing data was generally balanced across the three volume groups, the possibility of non-random missingness cannot be excluded, which may introduce attrition bias and affect the reliability of outcome estimates. Therefore, the reliability of the corresponding outcome assessments may be compromised and should be interpreted with caution. The study also focused on internal comparisons within a single technique and did not directly compare SP-SP with OSP, conventional RASP, or endoscopic enucleation procedures, so no conclusion can be drawn regarding relative superiority among treatment strategies. Finally, because part of the cohort overlapped with the learning phase, an interaction between learning-curve effects and volume effects cannot be excluded [8, 20]. However, sensitivity analyses excluding cases from the learning phase yielded broadly consistent results, supporting the robustness of the main findings.

## Conclusion

SP-SP appears to be a feasible and safe option for patients with very large prostates. Although larger prostate volume was associated with longer operative times and greater intraoperative blood loss in this cohort, these differences did not translate into higher complication rates, longer hospitalization, or worse functional outcomes. These observations provide preliminary evidence supporting the safety of SP-SP across different prostate volume strata. Multicenter studies with adequate sample sizes, longer postoperative follow-up, and step-specific procedural analysis are advisable to further evaluate the efficacy of SP-SP and to guide targeted technical refinements.

## Supplementary information

Sensitivity Analysis After Excluding the Learning Phase

## Data Availability

The data that support this study are not publicly available due to patient privacy and institutional ethical restrictions but are available from the corresponding author on reasonable request.
