# Supplementary material for: Feasibility and safety of single-port transvesical simple prostatectomy in patients with very large prostates: A volume-stratified analysis
Source: Prostate Cancer Prostatic Dis. 2026 May 28;29(4):854–61. doi: 10.1038/s41391-026-01121-8 (PMC13623653; doi:10.1038/s41391-026-01121-8)
Supplement: Supplementary file 1 [file 41391_2026_1121_MOESM1_ESM.docx]

| Supplementary Table S1. Baseline characteristics of patients in different prostate volume groups after excluding the learning phase. | | | | |
| --- | --- | --- | --- | --- |
|  | Prostate volume group | | |  |
| Characteristics | Group A (80–120 mL)  (N = 29) | Group B (120–160 mL)  (N = 15) | Group C (>160 mL)  (N = 19) | *p* value |
| Age, Median (IQR) (years) | 29 \| 63.0 (59.0, 71.0) | 15 \| 66.0 (62.0, 75.0) | 19 \| 67.0 (65.0, 73.0) | 0.064 |
| Race, n (%) |  |  |  | 0.312 |
| African American | 10 (34.5%) | 6 (40.0%) | 10 (52.6%) |  |
| Asian | 2 (6.9%) | 0 (0.0%) | 0 (0.0%) |  |
| Caucasian | 5 (17.2%) | 1 (6.7%) | 5 (26.3%) |  |
| Hispanic | 12 (41.4%) | 8 (53.3%) | 4 (21.1%) |  |
| BMI, Median (IQR) (kg/m^2^) | 29 \| 27.1 (22.8, 29.5) | 15 \| 28.1 (26.1, 34.4) | 19 \| 31.1 (23.8, 37.3) | 0.152 |
| Prostate size, Median (IQR) (mL) | 29 \| 97.0 (83.7, 100.0) | 15 \| 134.0 (127.0, 140.0) | 19 \| 243.0 (176.0, 310.0) | **<0.001** |
| Age adjusted CCI, n (%) |  |  |  | 0.939 |
| 0-3 | 21 (72.4%) | 10 (66.7%) | 13 (68.4%) |  |
| 4-5 | 7 (24.1%) | 4 (26.7%) | 4 (21.1%) |  |
| ≥6 | 1 (3.4%) | 1 (6.7%) | 2 (10.5%) |  |
| ASA grade, n (%) |  |  |  | 0.149 |
| 2 | 13 (44.8%) | 9 (60.0%) | 5 (26.3%) |  |
| 3 | 16 (55.2%) | 6 (40.0%) | 14 (73.7%) |  |
| Learning curve phase, n (%) |  |  |  | 0.843 |
| Proficiency phase | 17 (54.8%) | 7 (53.8%) | 12 (63.2%) |  |
| Consolidation phase | 14 (45.2%) | 6 (46.2%) | 7 (36.8%) |  |
| Abbreviations: BMI, body mass index; CCI, Charlson comorbidity index; ASA, American Society of Anesthesiologists. | | | | |

| Supplementary Table S2. Preoperative characteristics of patients in different prostate volume groups after excluding the learning phase | | | | |
| --- | --- | --- | --- | --- |
|  | Prostate volume group | | |  |
| Characteristics | Group A (80–120 mL)  (N = 29) | Group B (120–160 mL)  (N = 15) | Group C (>160 mL)  (N = 19) | *p* value |
| Preoperative PSA, (ng/mL) | 21 \| 6.0 (2.4, 10.0) | 13 \| 10.1 (3.6, 10.7) | 16 \| 12.0 (6.3, 22.9) | **0.026** |
| Median (IQR) |  |  |  |  |
| Preoperative hemoglobin, (g/dL) | 25 \| 13.7 (12.9, 14.9) | 14 \| 13.2 (12.6, 14.5) | 16 \| 14.1 (12.2, 14.5) | 0.418 |
| Median (IQR) |  |  |  |  |
| Preoperative PVR, (ml) | 24 \| 137.5 (62.0, 327.5) | 8 \| 99.0 (19.5, 413.5) | 13 \| 184.0 (100.0, 293.0) | 0.501 |
| Median (IQR) |  |  |  |  |
| Preoperative IPSS | 14 \| 22.5 (19.0, 26.0) | 9 \| 16.0 (11.0, 25.0) | 6 \| 17.5 (15.0, 18.0) | 0.161 |
| Median (IQR) |  |  |  |  |
| Preoperative QoL | 15 \| 5.0 (4.0, 5.0) | 12 \| 4.0 (3.0, 5.5) | 8 \| 4.0 (3.0, 4.0) | 0.106 |
| Median (IQR) |  |  |  |  |
| Preoperative indwelling catheter, n (%) |  |  |  | 0.522 |
| No | 18 (62.1%) | 7 (46.7%) | 9 (47.4%) |  |
| Yes | 11 (37.9%) | 8 (53.3%) | 10 (52.6%) |  |
| Preoperative prostate medication, n (%) |  |  |  | 0.861 |
| 5α-Reductase inhibitor | 1 (3.4%) | 1 (6.7%) | 0 (0.0%) |  |
| Alpha Blocker | 18 (62.1%) | 7 (46.7%) | 11 (57.9%) |  |
| Alpha Blocker + 5α-Reductase inhibitor | 6 (20.7%) | 4 (26.7%) | 5 (26.3%) |  |
| Anticholinergic | 0 (0.0%) | 1 (6.7%) | 0 (0.0%) |  |
| None | 4 (13.8%) | 2 (13.3%) | 3 (15.8%) |  |
| Prior abdominal surgery, n (%) |  |  |  | 0.109 |
| No | 25 (86.2%) | 9 (60.0%) | 13 (68.4%) |  |
| Yes | 4 (13.8%) | 6 (40.0%) | 6 (31.6%) |  |
| Preoperative prostate biopsy, n (%) |  |  |  | 0.536 |
| No | 19 (67.9%) | 8 (53.3%) | 10 (52.6%) |  |
| Yes | 9 (32.1%) | 7 (46.7%) | 9 (47.4%) |  |
| Preoperative urine culture, n (%) |  |  |  | 0.554 |
| Negative | 21 (72.4%) | 12 (80.0%) | 12 (63.2%) |  |
| Positive | 8 (27.6%) | 3 (20.0%) | 7 (36.8%) |  |
| Abbreviations: PSA, prostate-specific antigen; PVR, postvoid residual volume; IPSS, International Prostate Symptom Score; QoL, quality of life; MME, morphine milligram equivalents. | | | | |

| Supplementary Table S3. Intraoperative outcomes of patients in different prostate volume groups after excluding learning curve phase. | | | | |
| --- | --- | --- | --- | --- |
|  | Prostate volume group | | |  |
| Characteristics | Group A (80–120 mL)  (N = 29) | Group B (120–160 mL)  (N = 15) | Group C (>160 mL)  (N = 19) | *p* value |
| Operative time, (minutes) | 29 \| 168.0 (144.0, 190.0) | 15 \| 175.0 (168.0, 227.0) | 19 \| 218.0 (196.0, 275.0) | **<0.001** |
| Median (IQR) |  |  |  |  |
| Estimated blood loss, (mL) | 29 \| 50.0 (30.0, 100.0) | 15 \| 100.0 (50.0, 200.0) | 19 \| 180.0 (100.0, 200.0) | **0.005** |
| Median (IQR) |  |  |  |  |
| Intraoperative opioid dose, (mg MME) | 28 \| 12.8 (12.0, 15.0) | 13 \| 13.0 (6.8, 17.0) | 18 \| 14.3 (12.5, 17.5) | 0.395 |
| Median (IQR) |  |  |  |  |
| Bladder irrigation, n (%) |  |  |  | 0.140 |
| No | 28 (96.6%) | 12 (80.0%) | 17 (94.4%) |  |
| Yes | 1 (3.4%) | 3 (20.0%) | 1 (5.6%) |  |
|  | | | | |

| Supplementary Table S4. Postoperative outcomes of patients in different prostate volume groups after excluding learning curve phase. | | | | |
| --- | --- | --- | --- | --- |
|  | Prostate volume group | | |  |
| Characteristics | Group A (80–120 mL)  (N = 29) | Group B (120–160 mL)  (N = 15) | Group C (>160 mL)  (N = 19) | *p* value |
| Postoperative hemoglobin (g/dL) | 14 \| 13.1 (12.1, 13.9) | 9 \| 11.1 (9.9, 12.4) | 7 \| 12.0 (8.9, 12.8) | 0.071 |
| Median (IQR) |  |  |  |  |
| Hemoglobin drop (g/dL) | 14 \| 1.1 (-0.1, 1.6) | 9 \| 1.0 (0.4, 2.7) | 7 \| 1.8 (0.4, 2.7) | 0.276 |
| Median (IQR) |  |  |  |  |
| Pain score at postoperative | 29 \| 3.0 (1.0, 4.0) | 15 \| 3.0 (0.0, 4.0) | 19 \| 3.0 (0.0, 4.0) | 0.737 |
| Median (IQR) |  |  |  |  |
| Postoperative opioid dose, (mg MME) | 29 \| 6.1 (1.7, 10.0) | 13 \| 0.0 (0.0, 5.0) | 18 \| 5.0 (0.0, 8.5) | 0.149 |
| Median (IQR) |  |  |  |  |
| Total opioid dose on POD 0, (mg MME) | 29 \| 20.0 (12.5, 26.0) | 13 \| 17.0 (10.0, 25.5) | 18 \| 21.0 (17.5, 23.0) | 0.561 |
| Median (IQR) |  |  |  |  |
| Time to catheter removal, (days) | 29 \| 4.0 (4.0, 6.0) | 14 \| 5.0 (4.0, 7.0) | 19 \| 5.0 (4.0, 7.0) | 0.321 |
| Median (IQR) |  |  |  |  |
| Length of hospital stay, (IQR) (hours) | 29 \| 8.0 (7.0, 9.0) | 15 \| 9.0 (9.0, 10.0) | 19 \| 9.0 (8.0, 11.0) | 0.145 |
| Same Day Discharge, n (%) |  |  |  | 0.492 |
| No | 3 (10.3%) | 1 (6.7%) | 4 (21.1%) |  |
| Yes | 26 (89.7%) | 14 (93.3%) | 15 (78.9%) |  |
| OFA, n (%) |  |  |  | 0.133 |
| No | 22 (75.9%) | 6 (46.2%) | 10 (55.6%) |  |
| Yes | 7 (24.1%) | 7 (53.8%) | 8 (44.4%) |  |
| Analgesia at discharge, n (%) |  |  |  | 0.341 |
| Opioid | 1 (3.4%) | 0 (0.0%) | 0 (0.0%) |  |
| No opioid | 23 (79.3%) | 11 (78.6%) | 15 (83.3%) |  |
| Association | 0 (0.0%) | 0 (0.0%) | 2 (11.1%) |  |
| No | 5 (17.2%) | 3 (21.4%) | 1 (5.6%) |  |
| Postoperative complications, n (%) |  |  |  | 0.320 |
| No | 22 (71.0%) | 8 (61.5%) | 16 (84.2%) |  |
| Yes | 9 (29.0%) | 5 (38.5%) | 3 (15.8%) |  |
| Clavien-Dindo grade, n (%) |  |  |  | 0.773 |
| 1 | 3 (33.3%) | 1 (20.0%) | 0 (0.0%) |  |
| 2 | 6 (66.7%) | 4 (80.0%) | 3 (100.0%) |  |
| Readmission, n (%) |  |  |  | 0.086 |
| No | 29 (100.0%) | 13 (86.7%) | 18 (94.7%) |  |
| Yes | 0 (0.0%) | 2 (13.3%) | 1 (5.3%) |  |
| Postoperative urine culture, n (%) |  |  |  | 0.544 |
| Negative | 12 (57.1%) | 8 (72.7%) | 8 (72.7%) |  |
| Positive | 9 (42.9%) | 3 (27.3%) | 3 (27.3%) |  |
| Abbreviations: POD, postoperative day; MME, morphine milligram equivalents; OFA, opioid-free analgesia; | | | | |

| Supplementary Table 5. Pathological and follow-up outcomes of patients in different prostate volume groups after excluding learning curve phase. | | | | |
| --- | --- | --- | --- | --- |
|  | Prostate volume group | | |  |
| Characteristics | Group A (80–120 mL)  (N = 29) | Group B (120–160 mL)  (N = 15) | Group C (>160 mL)  (N = 19) | *p* value |
| Specimen weight, (g) | 29 \| 58.0 (40.0, 67.0) | 15 \| 80.0 (74.0, 89.0) | 19 \| 151.0 (130.0, 224.0) | **<0.001** |
| Median (IQR) |  |  |  |  |
| Resection ratio, (%) | 29 \| 61.0 (49.0, 72.0) | 15 \| 62.0 (57.0, 67.0) | 19 \| 60.0 (54.0, 76.0) | 0.683 |
| Median (IQR) |  |  |  |  |
| Postoperative pathology, n (%) |  |  |  | >0.999 |
| BPH | 27 (93.1%) | 14 (93.3%) | 17 (89.5%) |  |
| Cancer | 2 (6.9%) | 1 (6.7%) | 2 (10.5%) |  |
| PSA at last follow-up, (ng/mL) | 11 \| 0.6 (0.2, 3.1) | 6 \| 0.2 (0.1, 0.4) | 10 \| 0.6 (0.2, 1.0) | 0.163 |
| Median (IQR) |  |  |  |  |
| PVR at last follow-up, (ml) | 27 \| 10.0 (0.0, 29.0) | 13 \| 0.0 (0.0, 24.0) | 17 \| 10.0 (0.0, 20.0) | 0.545 |
| Median (IQR) |  |  |  |  |
| Postoperative IPSS | 21 \| 4.0 (2.0, 6.0) | 9 \| 5.0 (2.0, 6.0) | 13 \| 5.0 (3.0, 7.0) | 0.468 |
| Median (IQR) |  |  |  |  |
| Postoperative QoL | 20 \| 1.0 (1.0, 2.0) | 11 \| 1.0 (0.0, 2.0) | 8 \| 1.0 (0.0, 1.5) | 0.462 |
| Median (IQR) |  |  |  |  |
| Continence in 3 months, n (%) |  |  |  | >0.999 |
| No | 1 (3.4%) | 1 (6.7%) | 1 (5.6%) |  |
| Yes | 28 (96.6%) | 14 (93.3%) | 17 (94.4%) |  |
| Continence in 6 months, n (%) |  |  |  | 1.000 |
| No | 0 (0.0%) | 0 (0.0%) | 0 (0.0%) |  |
| Yes | 18(100%) | 8(100%) | 10(100%) |  |
| Abbreviations: PSA, prostate-specific antigen; PVR, postvoid residual volume; IPSS, International Prostate Symptom Score; QoL, quality of life; BPH, benign prostatic hyperplasia. | | | | |
